# Interaction of Bovine Peripheral Blood Polymorphonuclear Cells and *Leptospira* Species; Innate Responses in the Natural Bovine Reservoir Host

**DOI:** 10.3389/fmicb.2016.01110

**Published:** 2016-07-19

**Authors:** Jennifer H. Wilson-Welder, Ami T. Frank, Richard L. Hornsby, Steven C. Olsen, David P. Alt

**Affiliations:** Infectious Bacterial Diseases Research Unit, National Animal Disease CenterAmes, IA, USA

**Keywords:** *Leptospira*, serovar Hardjo, neutrophils, bovine host

## Abstract

Cattle are the reservoir hosts of *Leptospira borgpetersenii* serovar Hardjo, and can also be reservoir hosts of other *Leptospira* species such as *L. kirschneri*, and *Leptospira interrogans*. As a reservoir host, cattle shed *Leptospira*, infecting other animals, including humans. Previous studies with human and murine neutrophils have shown activation of neutrophil extracellular trap or NET formation, and upregulation of inflammatory mediators by neutrophils in the presence of *Leptospira*. Humans, companion animals and most widely studied models of Leptospirosis are of acute infection, hallmarked by systemic inflammatory response, neutrophilia, and septicemia. In contrast, cattle exhibit chronic infection with few outward clinical signs aside from reproductive failure. Taking into consideration that there is host species variation in innate immunity, especially in pathogen recognition and response, the interaction of bovine peripheral blood polymorphonuclear cells (PMNs) and several *Leptospira* strains was evaluated. Studies including bovine-adapted strains, human pathogen strains, a saprophyte and inactivated organisms. Incubation of PMNs with *Leptospira* did induce slight activation of neutrophil NETs, greater than unstimulated cells but less than the quantity from *E. coli* P4 stimulated PMNs. Very low but significant from non-stimulated, levels of reactive oxygen peroxides were produced in the presence of all *Leptospira* strains and *E. coli* P4. Similarly, significant levels of reactive nitrogen intermediaries (NO_2_) was produced from PMNs when incubated with the *Leptospira* strains and greater quantities in the presence of *E. coli* P4. PMNs incubated with *Leptospira* induced RNA transcripts of IL-1β, MIP-1α, and TNF-α, with greater amounts induced by live organisms when compared to heat-inactivated leptospires. Transcript for inflammatory cytokine IL-8 was also induced, at similar levels regardless of *Leptospira* strain or viability. However, incubation of *Leptospira* strains with bovine PMNs did not affect *Leptospira* viability as measured by limiting dilution culture. This is in contrast to previously reported results of innate inflammatory activation by *Leptospira* in human and other animal models, or the activation and interaction of bovine PMNs with *Escherichia coli* and other bacterial pathogens. While it could be hypothesized that variations in innate receptor recognition, specifically variance in toll-like receptor 2, could underlie the observed reduction of activation in bovine PMNs, additional studies would be needed to explore this possibility. Reduction in neutrophil responses may help to establish nearly asymptomatic chronic *Leptospira* infection of cattle. This study emphasizes the importance of studying host-pathogen relationships in the appropriate species as extrapolation from other animal models may be incorrect and confounded by differences in the host responses.

## Introduction

Leptospirosis is a disease caused by pathogenic spirochetes in the genus *Leptospira*, incorporating numerous species and over 200 serovars. A globally important zoonosis, leptospirosis is transmitted through contact with contaminated soil or water, and urine from infected mammals. Disease ranges from mild febrile illness with flu-like symptoms to acute severe disease with pulmonary, renal and hepatic complications, and may result in death in incidental hosts. However, chronically infected, reservoir hosts are generally asymptomatic with intermittent shedding of bacteria in urine. Worldwide, cattle are most commonly infected with *Leptospira borgpetersenii* serovar Hardjo. Leptospirosis infection is the leading cause of reproductive failure in cattle, and can result in weak/stillborn calves, reduced growth rates, and reduced milk production, all contributing to considerable economic loss to the cattle producer. Some cattle develop a chronic infection/shedding state and serve as a reservoir of infection for cattle and other incidental hosts including humans. Serovar Hardjo infection in incidental hosts, like humans or dogs, can result in acute disease (Blackmore and Schollum, 1982; Ryan et al., 1982; Zuerner et al., 2012).

Neutrophilia has been observed in acute leptospirosis infections in dogs, hamsters and humans (Ryan et al., 1982; Kobayashi, 2001; Libraty et al., 2007; Ganoza et al., 2010; Chow et al., 2012; De Silva et al., 2014) (D. Alt, unpublished observations). It is currently unknown if neutrophilia occurs in newly infected cattle before or during the onset of chronic disease. The impact of *Leptospira* infection on circulating neutrophils during infection in cattle remains uncharacterized.

Neutrophils are the first-line of defense for the innate immune system. Released as mature cells from the bone marrow, neutrophils are the most numerous leukocyte in blood (Nauseef and Borregaard, 2014). Circulating in an active state, neutrophils have the ability to quickly localize to specific tissues to combat infection (Nauseef and Borregaard, 2014). Neutrophils can participate in pathogen clearance or neutralization in a variety of ways including: production of azurophilic granules (which contain a number of proteolytic enzymes, elastase, antimicrobial defensins, and myeloperoxidase), production of antimicrobial peptides, formation of reactive oxygen and nitrogen species, production of cytokines which interact with other immune cells, phagocytosis of the pathogen, and formation of Neutrophil Extracellular Traps (NETs) (Amulic and Hayes, 2011). Previous reports have also suggested that *Leptospira* spp. are sensitive to bacterial killing by reactive oxygen intermediates such as H_2_O_2_ and low molecular weight primary granule components released from neutrophils (Murgia et al., 2002). Recently, it was shown that *Borrelia burgdorferi*, a spirochete that is the causative agent of Lyme's Disease, was ensnared and killed by human neutrophil NETs (Menten-Dedoyart et al., 2012). Furthermore, human neutrophils in culture with pathogenic *Leptospira interrogans* serovar Copenhageni produced NETs, reducing leptospiral viability (Scharrig et al., 2015). Humans, mice and hamsters all exhibit acute disease when infected with pathogenic *Leptospira* serovars. These authors also indicated that mice had nucleosomes, hallmarks of NET formation, circulating in blood after infection, hypothesizing that NET formation may play a role in prevention of bacterial dissemination (Scharrig et al., 2015). Thus, *Leptospira* should be significantly impaired by the innate abilities of neutrophils and other classical innate immune cells. The effect of bovine neutrophils or other innate immune cells on *Leptospira* has not been studied.

Neutrophils are recognized as an important contributor of cytokines and chemokines at the site of an inflammatory response. IL-8 has a potent effect on neutrophils themselves, as well as being the primary chemokine produced by neutrophils after contact with a foreign particle (Scapini et al., 2000; Walter and Morck, 2002). Depending on the stimulus, neutrophil derived cytokines can impact the magnitude and the duration of inflammation via production of IL-10, activation of T-helper cells, and by recruitment of other phagocytes to the inflammatory foci (Scapini et al., 2000; Thomas and Schroder, 2013). Bovine neutrophils have been shown to express IL-1β, IL-8, IL-10, IFN-γ, TNF-α, and MIP-1α in response to bacterial pathogens *in vitro* (Hassfurther et al., 1994; Scapini et al., 2000; Worku and Morris, 2009). During acute infection with *L. interrogans* serovar Icterohaemorrhagiae, hamsters upregulate gene expression of TNF-α, TGF-β, IP-10 (CXCL10), and IL-10 at the site of infection (kidney) and peripheral blood mononuclear cells upregulate expression of TNF-α, IFN-γ, IL-12 (Vernel-Pauillac and Merien, 2006; Lowanitchapat et al., 2010).

Attempts to understand the innate immune responses in cattle to *Leptospira* infection through extrapolation of results from human studies or those employing laboratory animal models using *Leptospira* strains typically associated with acute disease in humans is less than optimal (Vernel-Pauillac and Merien, 2006; Lowanitchapat et al., 2010; Fraga et al., 2011; Goris et al., 2011). Infection in cattle is commonly caused by highly host-adapted strains of *Leptospira* that primarily establish chronic infections in the kidneys and reproductive tract. While it has been previously reported that natural killer (NK) T cells and T helper type 1 (Th1) cells may play a role in adaptive immune responses to vaccination, studies evaluating the innate responses to leptospiral organisms during active infection in cattle are lacking (Naiman et al., 2002; Zuerner et al., 2011). Recent studies have highlighted differences in innate recognition of *Leptospira* between mouse and human cells (Fraga et al., 2011) and even greater differences exist between mice and cattle innate immune recognition (Werling et al., 2009; Bryant and Monie, 2012). It is unknown if bovine neutrophils are activated by *Leptospira* bacteria, or participate in the killing of host-adapted *Leptospira* strains in cattle. Therefore our studies were designed to characterize naïve bovine neutrophil activation after incubation various *Leptospira* strains. In contrast to studies in human and laboratory animal models, bovine neutrophils exhibited only modest or slight activation in response to incubation with *Leptospira* bacteria and showed no reduction in *Leptospira* viability. These results illustrate the complicated *Leptospira*-host relationships and underscore the necessity of studying both reservoir and acute hosts.

## Materials and methods

### Neutrophil preparation

All procedures involving the use of cattle were approved by the Animal Care and Use Committee at National Animal Disease Center Ames, IA under protocol numbers 2677 and 2357. Whole blood from adult female Jersey and Holstein cattle in the blood donor pool free of disease or discernable health problems were used for isolation of peripheral blood polymorphonuclear cells (PMNs). Animals were sero-negative by microscopic agglutinating antibody test (MAT) for all *Leptospira* serovars used in this study. Peripheral whole blood was collected by venipuncture in acid-citrate dextrose anticoagulant and processed within 1 h of collection. Whole blood was diluted 1:2 with sterile phosphate buffered saline (PBS, pH 7.2) and centrifuged at 1000 × g for 40 min at 25°C. Plasma and buffy coat (lymphocyte layer) were removed. Red blood cells (RBCs) were lysed by dilution of the red blood cell pellet 1:5 in 150 mM ammonium chloride 10 mM Tris lysis buffer for 1.5 min while rotating. Remaining cells were washed 3-4 times by centrifugation with PBS and resuspended in phenol red-free RPMI 1640 (GIBCO) supplemented with 10% heat inactivated to 70°C fetal bovine serum and 1 mM HEPES (hereafter referred to as cRPMI). Heat inactivation at 70°C for 1 h inactivates endogenous DNases that can interfere with NETosis assays (von Kockritz-Blickwede et al., 2009). Live cells were counted by trypan blue exclusion on a hemacytometer. To determine cell morphology and purity, 1 × 10^5^ cells were washed once by centrifugation, resuspended in PBS with 0.5% fetal bovine serum and applied to a glass slide using a Shandon Cytocentrifuge and Cytospin apparatus (Shandon Inc.). Slides were methanol-fixed, stained with Giemsa Stain and visually inspected for neutrophil percentage using a light microscope.

### Bacterial preparation

*L. borgpetersenii* serovar Hardjo strains 203 and JB197 were previously isolated from beef steers during slaughter (Miller et al., 1991). *L. interrogans* serovar Pomona type kennewicki isolate RM211 was originally isolated from a swine fetus (Thiermann et al., 1985). *L. interrogans* serovar Copenhageni strain Fiocruz L1-130 was a gift from Dr. David Haake (Los Angles Veteran Health Care System). All pathogenic *Leptospira* strains used in the current study were pathogenic in hamsters and used at no more than 3 *in vitro* passages from hamster infection. Non-pathogenic *Leptospira biflexa* strain Patoc (ATCC^®^ 23582™) was purchased from American Type Culture Collection. All *Leptospira* strains were propagated in modified T80/40/LH media with 5-fluorouracil (100 μg/ml) at 29°C as described previously (Zuerner, 2005; Zuerner et al., 2011, 2012). In some assays, *L. borgpetersenii* serovar Hardjo strain JB197 was inactivated by incubation at 56°C for 1 h with bacterial inactivation (killing/no growth) confirmed by culture. *Escherichia coli* strain P4 was a gift from Dr. John Lippolis (Ruminant Diseases and Immunology Research Unit, National Animal Disease Center, Ames IA). *E. coli* P4 was grown overnight in tryptic soy broth with 1% fetal bovine serum. Log phase bacteria were quantified by direct counting under dark field using a Petroff-Hauser counting chamber, washed twice by centrifugation in PBS and resuspended in cRPMI. If needed, *E*. *coli* were held on ice until incubation with neutrophils.

### Observation of neutrophil extracellular traps (NETs)

Following methods similar to previously published studies (Lippolis et al., 2006; Fuchs et al., 2007; Aulik et al., 2010; Wardini et al., 2010), bovine PMNs were seeded at 1 × 10^6^ cells per ml onto 8 well chamber slides (Lab-Tek). Cells were allowed to settle for 1 h at 37°C under 5% CO_2_, before bacteria or other stimulants were added. *Leptospira* and *E. coli* strain P4 were added at a multiplicity of infection (MOI) of 10 bacterial cells per PMN. Positive control stimulant consisted of Phorbol 12-myristate 13-acetate (PMA) (Sigma) at a final concentration of 25 ng/ml. After addition of bacteria or stimulant, slides were incubated at 37°C for 2 h. To visualize NETS, cells were centrifuged gently at 400 × g for 5 min at 25°C, and supernatant aspirated. LIVE/DEAD^®^ *Bac*Light™ Bacterial Viability and Counting Kit (Molecular Probes) stain was used per manufacturer's recommendation with the following modification: Red:Green dye ratios were mixed in 2:1 proportion of component A to component B, diluted 1:50 in PBS. 100 μl was applied to each well of the slide and incubated in the dark for 15 min at room temperature. Stain was aspirated and slides rinsed with 500 μl PBS by careful application and removal using a pipette. Chambers were removed and coverslips were mounted with included oil component from *Bac*Light kit and visualized within 48 h. Slides were visualized using a Nikon E8400 microscope, 40x and 60x objectives, triple fluorescence filter and digital camera attachment. Three representative fields were imaged per slide.

### NET quantitation

Following method published in Chuammitri et al. (2009), isolated bovine PMNs were plated at 1 × 10^6^ in quadruplicate in 96 well plates for NET stimulation and quantitation. Cells were allowed to settle in plates for 1 h at 37°C, 5% CO_2_, at which time bacteria MOI 10 or stimulant was added to quadruplicate wells and incubated at 37°C for 2 h. Replicate wells were set up in the same manner, with the addition of 100 U/ml DNase I (RQ1 RNase free-DNase, Promega). After incubation, micrococcal nuclease (Worthington Biochemical Corporation) was added to a final concentration of 0.1 U/ml and incubated for an additional 30 min at 37°C. 5 mM EDTA was added to stop the reaction. Plates were centrifuged at 400 × g for 2.5 min at 25°C, and 100 μl of supernatant removed. DNA concentration of the supernatant was quantified (PicoGreen DNA quantification kit, Molecular Probes) and read in a fluorescence plate reader (SpectraMAX GeminiXS, Molecular Devices, excitation 492 nm, emission 520 nm; with SOFTMax PRO software). Data is expressed as mean of replicate wells ng/ml DNA in supernatant. Data was analyzed from three independent experiments using cells isolated from six animals. Data was analyzed using Graphpad Prism 6 Software and one-way ANOVA with Tukey's multiple comparisons post-test.

### NET confirmation

To confirm that the extracellular structures observed visually and by quantification of DNA in cellular supernatant were indeed NETs, bovine PMNs were seeded at 1 × 10^6^ cells per ml onto 8 well chamber slides (Lab-Tek). Cells were allowed to settle for 1 h at 37°C under 5% CO_2_, before bacteria or other stimulants were added. *Leptospira* and *E. coli* strain P4 were added at a multiplicity of infection (MOI) of 10 bacterial cells per PMN. Positive control stimulant consisted of PMA at a final concentration of 25 ng/ml. After addition of bacteria or stimulant, slides were incubated at 37°C for 2 h. After incubation, slides were centrifuged at 400 × g for 5 min at 25°C and supernatant carefully aspirated. Slides were fixed by adding 0.5 ml of 4% formalin in PBS to each chamber for 10 min at room temperature. Fixative was carefully aspirated, chamber well rinsed with PBS, and 0.5 ml PBS containing 1% normal goat serum was added (Equitech-Bio Inc.) and 10 μl azide-free Fc Receptor Blocker (INNOVEX Biosciences). After overnight incubation in humidified chamber at 4°C, chamber portion of the slide was removed and slides were stained with anti-bovine H2A antibody (AbCam rabbit polyclonal to Histone H2A, ab13923, 1:200 dilution), followed by Alexa Fluor 594 F(ab)'2 goat anti-rabbit (1:2000 dilution) and DAPI nuclear counterstain (Molecular Probes, 1:3000 dilution). Slides were visualized using a Nikon E8400 microscope at 40x magnification with triple fluorescence filter and digital camera attachment. The total number of cells and number of cells with H2A staining were hand counted for five random fields. Data is presented as the mean percentage of H2A positive PMN cells from 4 different cows assayed in two independent experiments. Data was analyzed using Graphpad Prism 6 Software and one-way ANOVA with Tukey's multiple comparisons post-test.

### Production of reactive oxygen species (ROS) by PMNs

As a surrogate reactive oxygen intermediate activity in the inflammatory process, ROS in the form of H_2_O_2_ was measured in cells and cell supernatants after stimulation with *Leptospira*. Cells and supernatants from NET quantitation experiments described for NET quantitation were stored at −80°C. Cells were lysed by freeze-thaw fracture and lysis confirmed by visual inspection. Peroxide concentration was determined using Amplex Red Reagent Kit (Molecular Probes) following manufacturer's recommendations. Data presented is the mean results from quadruplicate wells of cells isolated from six individual cows on two separate occasions. Data was analyzed using Graphpad Prism 6 Software and one-way ANOVA with Tukey's multiple comparisons post-test.

### Production of reactive nitrogen species (RNS) by PMNs

Cells and supernatants from NET quantitation experiments described above were stored at −80°C. Cells were lysed by freeze-thaw fracture and lysis confirmed by visual inspection. Nitrate concentration was determined using Griess Reagent Kit (Molecular Probes) following manufacturer's recommendations. Data presented is the mean results from quadruplicate wells of cells isolated from six individual cows on two separate occasions. Data was analyzed using Graphpad Prism 6 Software and one-way ANOVA with Tukey's multiple comparisons post-test.

### Cytokine gene transcription by activated PMNs

Isolated PMNs were plated in 24-well plates at a concentration of 2.5 × 10^6^/ml. Cells were allowed to settle in plates for 30 min at 37°C, 5% CO_2_, at which time bacteria (MOI 10) or stimulant were added to triplicate wells. Plates were incubated for 1.5 h at 37°C. After incubation, plates were centrifuged for 7 min at 500 × g at 25°C, and supernatants removed. RNA was extracted from the cell pellet (RNeasy + Mini Column Kit, Qiagen) according to manufacturer's protocol. RNA quality was assessed using Agilent 2100 Bioanalyzer. cDNA was synthesized using Invitrogen Superscript III polymerase, oligo dT, dNTPs, 0.1M DTT, and RNAse H. Real-Time PCR was performed using Qiagen 2X SYBR Green QuantiFast kit on an ABI 7900 384-well platform. 6 μl of cDNA template in a 20 μl reaction using primers listed in Table 1 with the following amplification conditions: 2 min at 50°, 10 min at 95°, 40 cycles of 15 s at 95° and 1 min at 60°, and a dissociation step (15 s at 95°, 1 min at 60°, 15 s at 95°, 15 s at 60°). Data was analyzed by 2^(−Δ*ΔCT*)^ method as outlined in Livak and Schmittgen (2001) with no stimulation being the control treatment and normalized to housekeeping gene RPS9. All statistical calculations were performed on log_2_ transformed data and presented as means of 4 animals tested in two independent experiments.

**Table 1:** **Bovine RT-PCR Primers Used**.

**Gene**	**Primer**	**Direction**	**References**
RPS9	CGC CTC GAC CAA GAG CTG AAG	Forward	McGill et al., 2013
	CCT CCA GAC CTC ACG TTT GTT CC	Reverse	
IL-1β	GTG ACG AGA ATG AGC TGT TAT TTG	Forward	Zuerner et al., 2007
	TGT TGT AGA ACT GGT GAG AAA TCT G	Reverse	
MIP-1α	AAG CCT GGT GTC ATC TTC C	Forward	McGill et al., 2013
	CTC CAG GTC GGT GAT GTA TTC	Reverse	
TNF-α	TCT ACC AGG GAG GAG TCT TCC A	Forward	Zuerner et al., 2007
	GTC CGG CAG GTT GAT CTC A	Reverse	
TGF-β	CTG AGC CAG AGG CGG ACT AC	Forward	McGill et al., 2013
	TGC CGT ATT CCA CCA TTA GCA	Reverse	
IL-17	CAC AGC ATG TGA GGG TCA AC	Forward	Vordermeier et al., 2009
	GGT GGA GCG CTT GTG ATA AT	Reverse	
IFN-γ	AGA ATC TCT TTC GAG GCC GGA G	Forward	McGill et al., 2013
	TAT TGC AGG CAG GAG GAC CAT TAC	Reverse	
IL-8	GTG TGA AGC TGC AGT TCT GTC	Forward	Zuerner et al., 2007
	GGT GGA AAG GTG TGG AAT GTG	Reverse	

### Bacterial killing by PMNs

PMNs were seeded at 1 × 10^6^ in triplicate in 96 well plates and allowed to settle for 1 h at 37°C, 5% CO_2_. *Leptospira* or *E. coli* P4 were added at an MOI of 10 and plates were centrifuged at 400 × g for 5 min at 25°C. After incubation for 4 h at 37°C, PMNs were lysed by addition of sterile filtered Saponin (0.05% final concentration). *Leptospira* samples were cultured by limiting dilution in modified T80/40/LH (Tween 80/Tween 40/ lactalbumin hydrolysate) with 5-fluorouracil (100 μg/ml) and incubated at 29°C for up to 4 weeks (Goris et al., 2011). Tubes were inspected visually for evidence of growth by dark field microscopy. Samples from PMNs incubated with *E. coli* P4 were serially diluted in PBS and plated on tryptic soy agar supplemented with 1% yeast extract and 5% sheep blood. Bacterial numbers were determined by plate counts after incubation at 37°C for 48 h. Data presented as percent survival (bacterial survival after incubations with PMNs divided by bacteria counts after incubation in the absence of PMNs) (Goris et al., 2011). Data presented is representative of duplicate independent experiments utilizing cells isolated from six animals.

### Effect of immune serum on PMN activation

After counting, cells were divided equally into four portions and resuspended in cRPMI supplemented with 20% bovine serum archived from a previous experiment (Zuerner et al., 2011). The study used a commercial vaccine containing *L. borgpetersenii* serovar Hardjo (type hardjo-bovis) (http://pfizerah.com) with 2 doses given 4 weeks apart and animals were challenged (1 year after vaccination) with *L. borgpetersenii* serovar Hardjo strain 203. Serum was selected to provide the following groups: (1) naïve cattle, (2) cattle receiving a commercial leptospirosis vaccine preparation 12 weeks post-vaccination (Vaccinated), (3) naïve cattle 8 weeks post-infection with *L. borgpetersenii* serovar Hardjo strain 203 (Challenged), and (4) cattle receiving a commercial leptospirosis vaccine and challenged with *L. borgpetersenii* serovar Hardjo strain 203, 8 weeks post-challenge (Vaccinated + Challenged) (Zuerner et al., 2011). Serum was heat inactivated to destroy endogenous nucleases and pooled serum titer was verified by MAT performed as previously reported (Zuerner et al., 2011). Resulting MAT titers to all *Leptospira* strains in this study are given in Table S1. Net quantitation and bacterial survival/killing studies were conducted with immune serum incubated PMNs as described above.

## Results

### Visualization, quantification, and confirmation of bovine PMN net formation following incubation with leptospira

**Figure 1:**
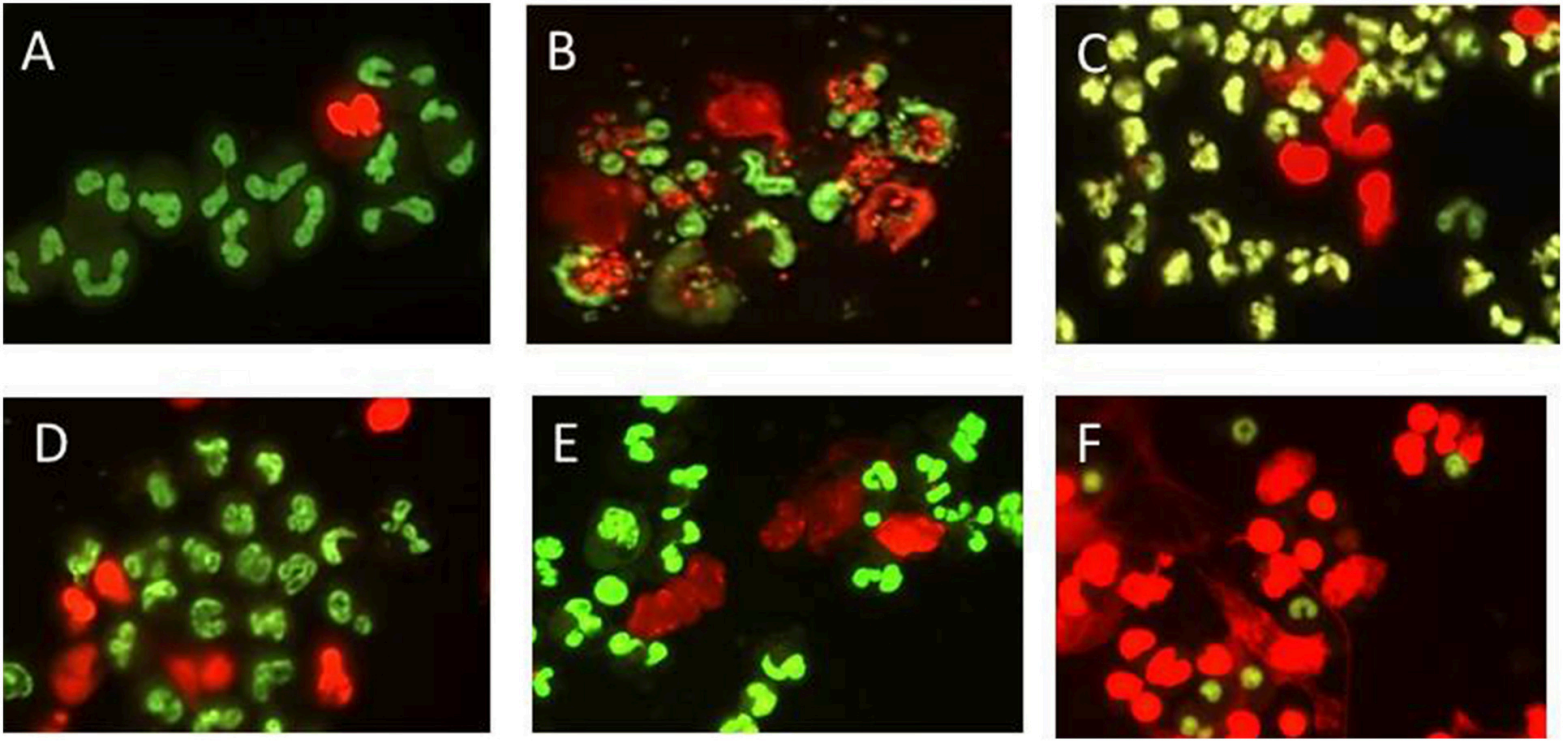
**Representative visualization of bovine PMN NET formation using DNA binding dye that is either cell permeable (green) or impermeable (red) indicating extrusion of cellular DNA or otherwise compromised membrane**. PMNs were incubated in chamber-slides with MOI 10 for 2 h in supplemented RPMI 1640 with 10% FCS, stained, mounted, and visualized using Nikon E8400 microscope 40x objective and digital camera attachment. **(A)** No Stimulant, **(B)** *E. coli* P4, **(C)** *L. borgpetersenii* serovar Hardjo strain JB197, **(D)** *L. borgpetersenii* serovar Hardjo strain 203, **(E)** *L. interrogans* serovar Pomona strain RM211, **(F)** PMA at a final concentration of 25 ng/ml.

After visualization with cell permeable and impermeable nucleic acid dyes (Figure 1), extracellular cloud-like formations consistent with previous reports of NETS could be observed (Brinkmann et al., 2004; Lippolis et al., 2006; Aulik et al., 2010; Wardini et al., 2010). *E. coli* strain P4 had been shown previously to induce bovine neutrophils to undergo NETosis and was included in all assays as a positive control (Lippolis et al., 2006). While there appeared to be an increase in the numbers of cells stained with the permeable dye when PMNs were incubated with *Leptospira* (Figures 1C–E), *E. coli* P4 (Figure 1B) or PMA (Figure 1F) over the unstimulated PMNs per field of view (Figure 1A), these results could not be quantitated due to uneven distribution of cells on the slide, variability across individual slides and variability from experimental day to day. While *E. coli* cells could be visualized in association with the PMNs (using 60x objective, not shown), we were unable to distinguish the thin, spirochetal shaped *Leptospira* from NETs or cellular membranes.

**Figure 2:**
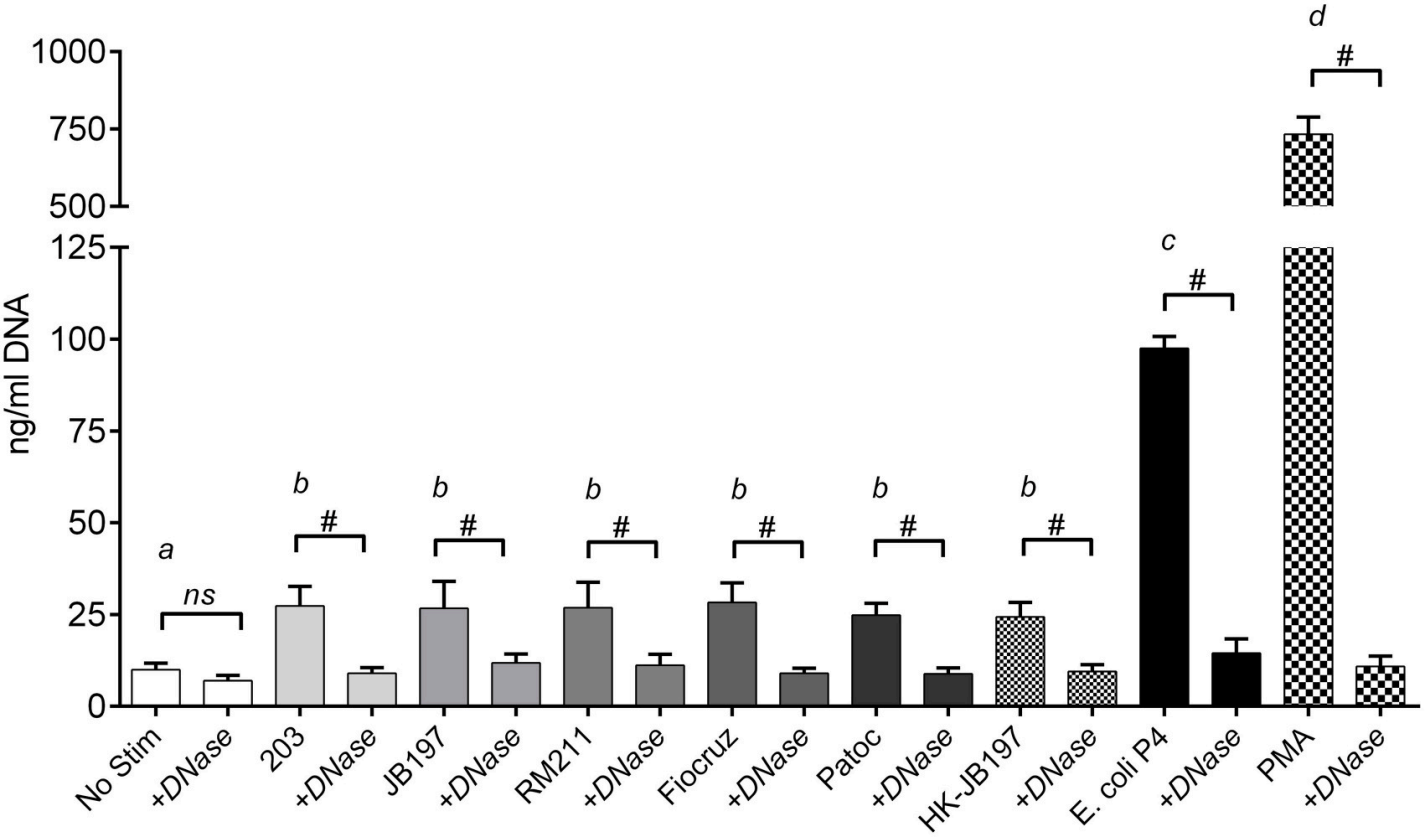
**Quantitation of extracellular DNA as an indication of NET formation**. Following incubation of isolated bovine PMNs in supplemented RPMI 1640 with 10% FCS, with media only (No Stimulant), *L. borgpetersenii* serovar Hardjo strain 203, *L. borgpetersenii* serovar Hardjo strain JB197, *L. interrogans* serovar Pomona strain RM211, *L. interrogans* serovar Copenhageni strain Fiocruz L1-130, *L. biflexa* strain Patoc, heat killed (HK) *L. borgpetersenii* serovar Hardjo strain JB197, or *E. coli* P4 in 96 well plates, extracellular DNA was released by digestion with Staphylococcal Endonuclease and supernatants assayed using fluormetric DNA quantitation assay (PicoGreen). Results depicted are mean and standard deviation (error bars) of six individual animals assayed on at least three different days without and with 100 U/ml DNase I (+DNase). Capped bars (without and with DNase) were compared to each other using students *T*-test # = *p* < 0.0001. Treatment bars (without +DNase) were compared to each other by one-way ANOVA with Tukey's multiple comparisons post-test. Treatments with different letters (*a, b, c, d*) are statistically different from each other but not from treatments with the same letter (*p* < 0.05).

Extracellular DNA extruded as part of NETosis was quantified in accordance with previous reports by quantifying DNA in culture supernatants (Lippolis et al., 2006; Aulik et al., 2010; Wardini et al., 2010). PMNs incubated with *E. coli* P4, demonstrated significant increases (*p* < 0.0001) of 97.5 ng/ml DNA in culture supernatant over unstimulated cells (10 ng/ml DNA in culture supernatant) as did the PMA stimulant (733.6 ng/ml DNA in culture supernatant) (Figure 2). Incubation with *Leptospira* strains induced approximately a two-fold increase in supernatant DNA concentration compared to non-stimulated PMNs resulting in strain 203.27.3 ng/ml, JB197 26.6 ng/ml, RM211 26.8 ng/ml, Fiocruz 28.3 ng/ml, Patoc 24.8 ng/ml, heat-killed JB197 24.3 ng/ml which were all statistically different from no stimulant, *E. coli* P4 and PMA (*p* < 0.0001) but not among the *Leptospira* strains (*p* < 0.05). These increases were eliminated by the inclusion of DNase in the assay (+DNase range 7 to 14.5 ng/ml).

**Figure 3:**
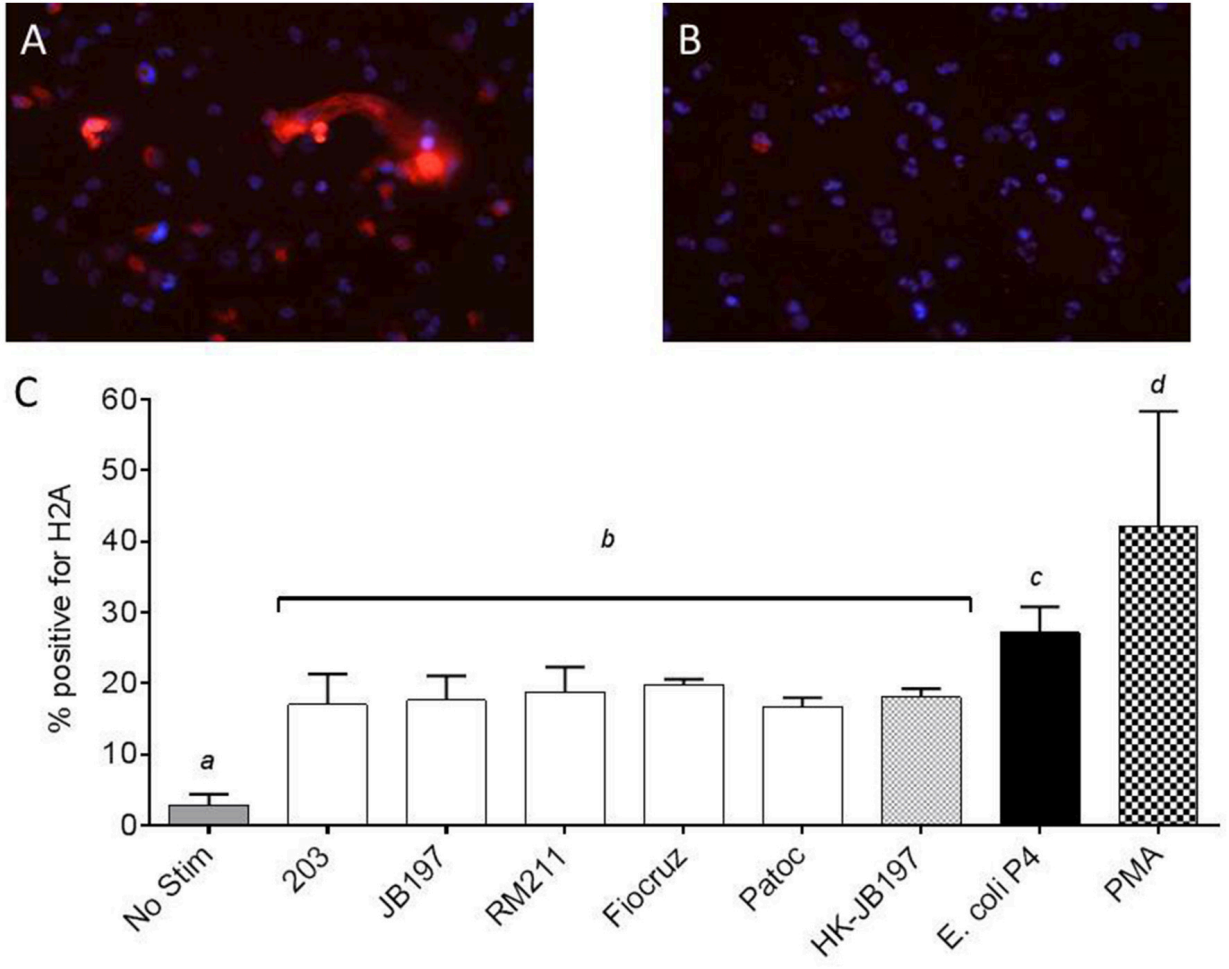
**Extracellular DNA has histone proteins consistent with NETosis of neutrophils**. PMNs were incubated in chamber-slides with media only (No Stimulant), *L. borgpetersenii* serovar Hardjo strain 203, *L. borgpetersenii* serovar Hardjo strain JB197, *L. interrogans* serovar Pomona strain RM211, *L. interrogans* serovar Copenhageni strain Fiocruz L1-130, *L. biflexa* strain Patoc, heat killed (HK) *L. borgpetersenii* serovar Hardjo strain JB197, *E. coli* P4, each at MOI 10, and PMA at a final concentration of 25 ng/ml for 2 h, stained with anti-bovine H2A antibody and DAPI nuclear counterstain, mounted and visualized using Nikon E8400 microscope using 40x objective. **(A)** Example of PMA stimulated bovine PMNs with positive extracellular staining for H2A. **(B)** Example of unstimulated bovine PMNs with negative extracellular staining for H2A **(C)** Mean and standard error of the mean (error bars) of four individual cows and two independent experiments as percentage of total number of cells in three random microscope fields staining positive for H2A. Means were compared to each other by one-way ANOVA with Tukey's multiple comparisons post-test. Treatments with different letters (*a, b, c, d*) are statistically different from each other but not from treatments with the same letter (*p* < 0.05).

To confirm that the extracellular structures visualized and quantified were indeed NETs, structures were labeled using an anti-Histone antibody (Gunderson and Seifert, 2015). Examples of positive and negative stained images are shown in Figures 3A,B, respectively. Of the *Leptospira* co-cultured cells, strain 203 17.0%, JB197 17.6%, RM211 18.7%, Fiocruz 19.8% Patoc 16.8%, heat-killed JB197 18.0% of total cells expressed NETs and were positive for H2A antibody staining (Figure 3C). 27.2% of the total cells cultured with *E. coli* P4 and 42.1% of the cells cultured with PMA were positive for NET-like formations and H2A antibody staining as described in Material and Methods.

### Incubation of bovine PMN with *leptospira* resulted in production of reactive oxygen species (ROS) or reactive nitrogen species (RNS)

**Figure 4:**
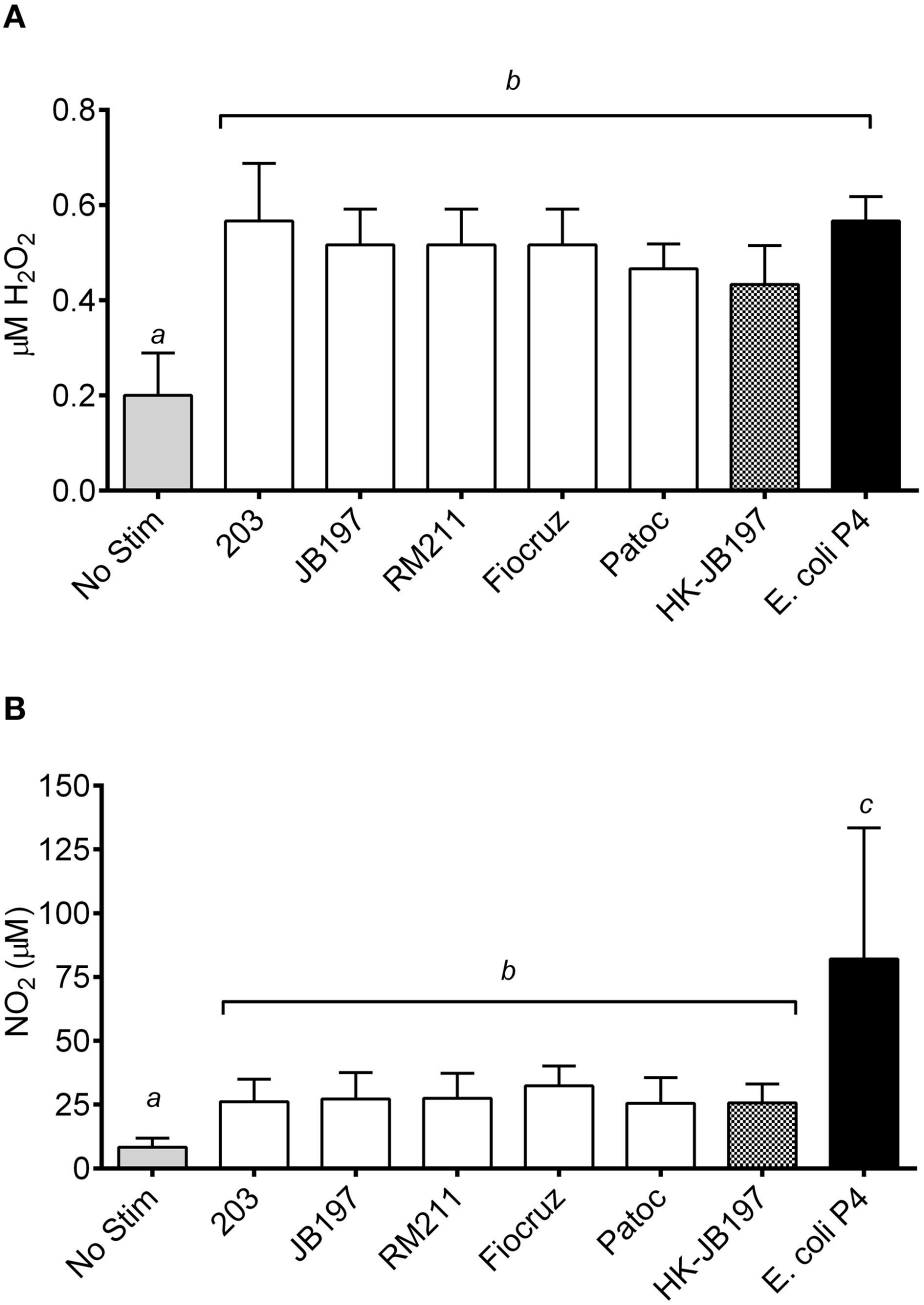
**Quantification of cellular (A) reactive oxygen species in the form of hydrogen peroxides and (B) reactive nitrogen species in the form of nitrates**. Following incubation of isolated bovine PMNs with media only (No Stim), *L. borgpetersenii* serovar Hardjo strain 203, *L. borgpetersenii* serovar Hardjo strain JB197, *L. interrogans* serovar Pomona strain RM211, *L. interrogans* serovar Copenhageni strain Fiocruz L1-130, *L. biflexa* strain Patoc, heat killed (HK) *L. borgpetersenii* serovar Hardjo strain JB197, or *E. coli* P4 in 96 well plates, cells and supernatant were stored at –20°C until assayed. **(A)** H_2_O_2_ and **(B)** NO_2_ production in cells and supernatant was determined by incubation with Amplex Red or Griess Reagent and optical density compared to standard following manufacturer's protocols. Results depicted are mean and standard error of the mean (error bars) of PMNs isolated from four individual cows assayed in three independent experiments. Means were compared to each other by one-way ANOVA with Tukey's multiple comparisons post-test. Treatments with different letters (*a, b, c*) are statistically different from each other but not from treatments with the same letter (*p* < 0.05).

In an effort to determine if ROS and RNS were generated, PMNs and culture supernatants were tested for levels of peroxide and nitrates. Bovine PMNs produced similar quantities of H_2_0_2_ when stimulated with *Leptospira* strains (strain 203 0.6 μM H_2_0_2_, JB197 0.5 μM H_2_0_2_, RM211 0.5 μM H_2_0_2_, Fiocruz 0.5 μM H_2_0_2_, Patoc 0.5 μM H_2_0_2_, heat killed JB197 0.4 μM H_2_0_2_) or *E. coli* P4 (0.6 μM H_2_0_2_,) (Figure 4A). While all were statictically significant (*p* < 0.05) from No Stimulant (0.2 μM H_2_0_2_,) it is unknown if the low levels of H_2_0_2_ observed (0.4–0.6 μM) has any biological relevance. In contrast, bovine PMNs incubated with *E. coli* P4 induced significantly (*p* < 0.05) higher levels of nitrates (82.1 μM NO_2_) in comparison to cells stimulated with *Leptospira* strains (Figure 4B). While there was no statistical difference between the cattle-adapted (strain 203 26.2 μM NO_2_, JB197 27.2 μM NO_2_), other pathogenic *Leptospira* strains (RM211 27.7 μM NO_2_, Fiocruz 32.3 μM NO_2_) or non-pathogenic *Leptospira* (Patoc 25.5 μM NO2, or heat-killed JB197 25.7 μM NO_2_), all were greater and significant (*p* < 0.05) than cells receiving no stimulation (8.4 μM NO_2_) (Figure 4B).

### Induction of inflammatory cytokine gene transcripts

**Figure 5:**
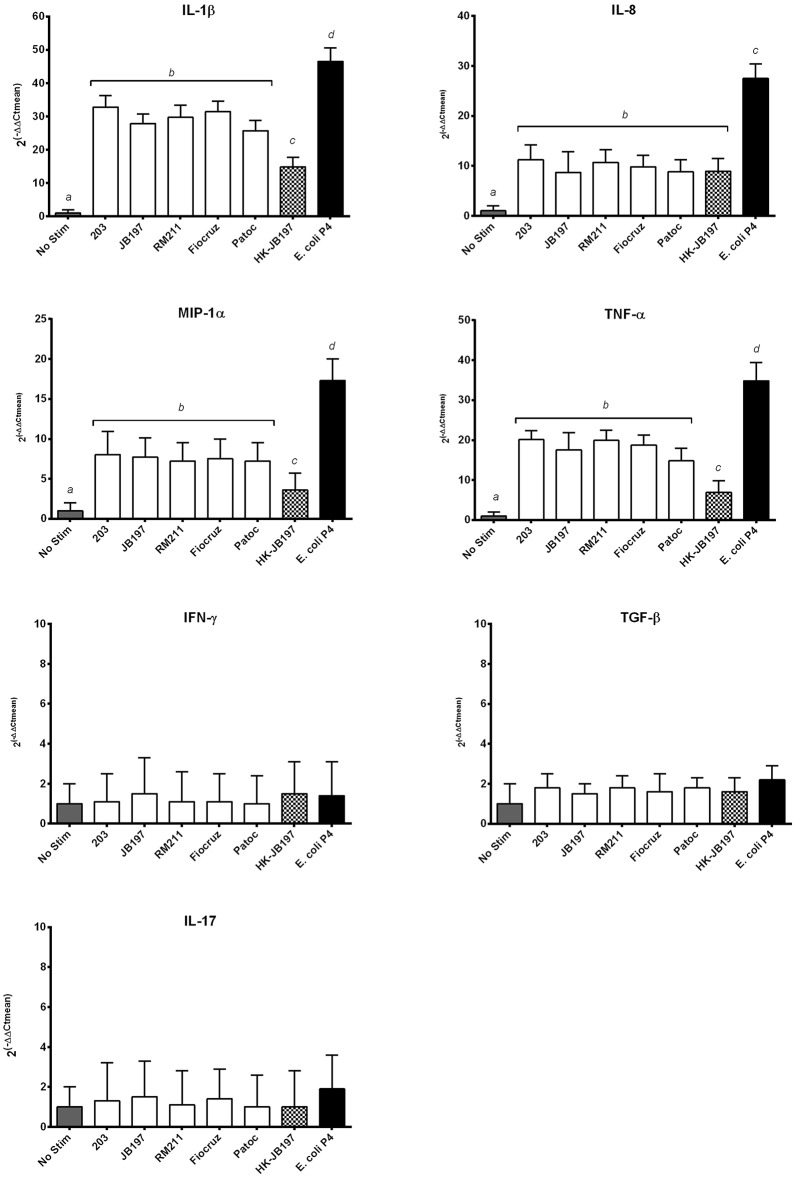
*****Leptospira*** induce inflammatory cytokine mRNA transcript expression**. Bovine PMNs were incubated with media only (No Stim), *L. borgpetersenii* serovar Hardjo strain 203, *L. borgpetersenii* serovar Hardjo strain JB197, *L. interrogans* serovar Pomona strain RM211, *L. interrogans* serovar Copenhageni strain Fiocruz L1-130, *L. biflexa* strain Patoc, heat killed (HK) *L. borgpetersenii* serovar Hardjo strain JB197, or *E. coli* strain P4 for 1.5 h at MOI 10. The relative mRNA expression of cytokines IL-1β, IL-8, TNF-α, MIP-1α, TNF-α, IFN-γ, TGF-β, and IL-17 were assayed by quantitative RT-PCR. Data reported as means and standard error of the mean (error bars) for PMNs isolated from 4 individual naive cows assayed in two independent experiments. Data is depicted as 2^(−Δ*ΔCt*)^ where cytokine expression is relative increase over No Stim normalized to housekeeping gene RPS9. Statistical calculations were performed on log_2_ transformed data. Treatments with different letters (*a, b, c, d*) are statistically different from each other but not from treatments with the same letter (*p* < 0.05).

Neutrophils can influence local inflammation by production of pro-inflammatory cytokines. RNA was extracted from bovine PMNs incubated for 1.5 h with *Leptospira* strains, heat killed *Leptospira* cells or *E. coli* P4. Cytokine gene expression for each *Leptospira* strain or *E. coli* strain P4 was compared to No Stimulant and normalized to housekeeping gene RSP9 by qRT-PCR. For IL-1β, IL-8, MIP-1α, and TNF-α, gene expression was significantly increased for all treatments above no stimulant or background levels, with *E. coli* P4 being greater than any of the *Leptospira* strains tested (Figure 5). Interestingly, for IL-1β, MIP-1α, and TNF-α heat-killed JB197 induced relatively less expression than cattle adapted (203, JB197), pathogenic (RM211, Fiocruz), or the live saprophyte (Patoc) strain, but was still significantly above no stimulant (*p* < 0.05). 2^(−Δ*ΔCT*)^ values for IL-1β relative gene expression were 1, 32.8, 27.8 29.7, 31.4, 24.7, 15.8, and 46.4 for No Stim, 203, JB197, RM211, Fiocruz, Patoc, heat-killed JB197, and *E. coli* P4 respectively. 2^(−Δ*ΔCT*)^ values for IL-8 relative gene expression were 1, 11.2, 8.7, 10.7, 9.8, 8.8, 8.9, and 25.5 for No Stim, 203, JB197, RM211, Fiocruz, Patoc, heat-killed JB197, and *E. coli* P4 respectively. 2^(−Δ*ΔCT*)^ values for MIP-1α relative gene expression were 1, 8.0, 7.7, 7.2, 7.5, 7.2, 3.6, and 17.3 for No Stim, 203, JB197, RM211, Fiocruz, Patoc, heat-killed JB197, and *E. coli* P4 respectively. 2^(−Δ*ΔCT*)^ values for TNF-α relative gene expression were 1, 20.1, 17.5 19.9, 18.7, 14.8, 6.9, and 34.8 for No Stim, 203, JB197, RM211, Fiocruz, Patoc, heat-killed JB197, and *E. coli* P4, respectively. Cytokine gene expression showed no increase in gene expression of TGF-β, IL-17 or IFN-γ above no stimulation or background levels, with all means being at or below 2.

### Incubation of bovine PMNs with leptospira did not reduce bacterial viability

**Figure 6:**
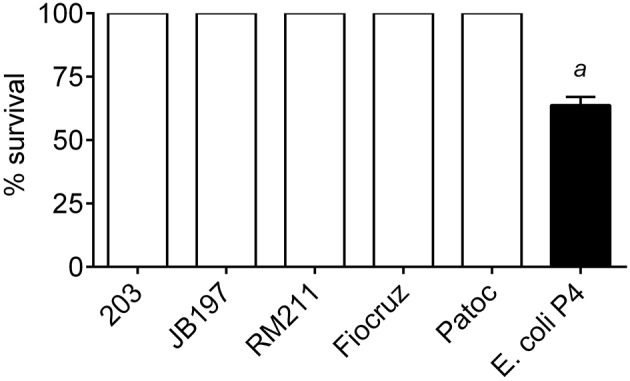
**Bacterial survival after incubation with bovine PMNs**. *L. borgpetersenii* serovar Hardjo strain 203, *L. borgpetersenii* serovar Hardjo strain JB197, *L. interrogans* serovar Pomona strain RM211, *L. interrogans* serovar Copenhageni strain Fiocruz L1-130, *L. biflexa* strain Patoc, or *E. coli* strain P4 were incubated with freshly isolated bovine peripheral blood polymorphonunclear cells (PMNs) at MOI 10 for 4 h. At the end of the incubation, PMNs were lysed by the addition of 0.05% Saponin. *Leptospira* were cultured for up to 4 weeks in serial dilution series tubes for estimation of number. *E. coli* P4 was diluted by serial dilution and spread on sheep blood supplemented tryptic soy agar plates and incubated overnight. Results depicted as percentage survival calculated from lowest dilution tube (or plate CFU count for *E. coli* P4) with positive growth compared to bacterial cells under the same incubation conditions (culture media, time, saponin, etc.) without the addition of bovine PMNs. Each bacterial strain was incubated with PMNs from four individual cows in duplicate sampled in two independent experiments. *a*: *E. coli* P4 was statically different from all *Leptospira* (63 vs. 100% survival; *p* < 0.05).

*Leptospira* strains and *E.coli* P4 (MOI 10) were incubated for 4 h with bovine PMNs. Following this incubation, PMNs were lysed with saponin, and bacterial viability determined by either limiting dilution culture in semi-solid media tubes for *Leptospira* or dilution and plating to obtain CFU for *E. coli* P4. Bacteria incubated under the same conditions but in the absence of PMNs were set as 100% survival. Incubation of the *Leptospira* with bovine PMNs had no effect on *Leptospira* viability (*p* < 0.05), however PMNs had a negative impact on *E. coli* P4 viability, resulting in 64% survival (Figure 6).

### Effect of immune serum on PMN NETosis

**Figure 7:**
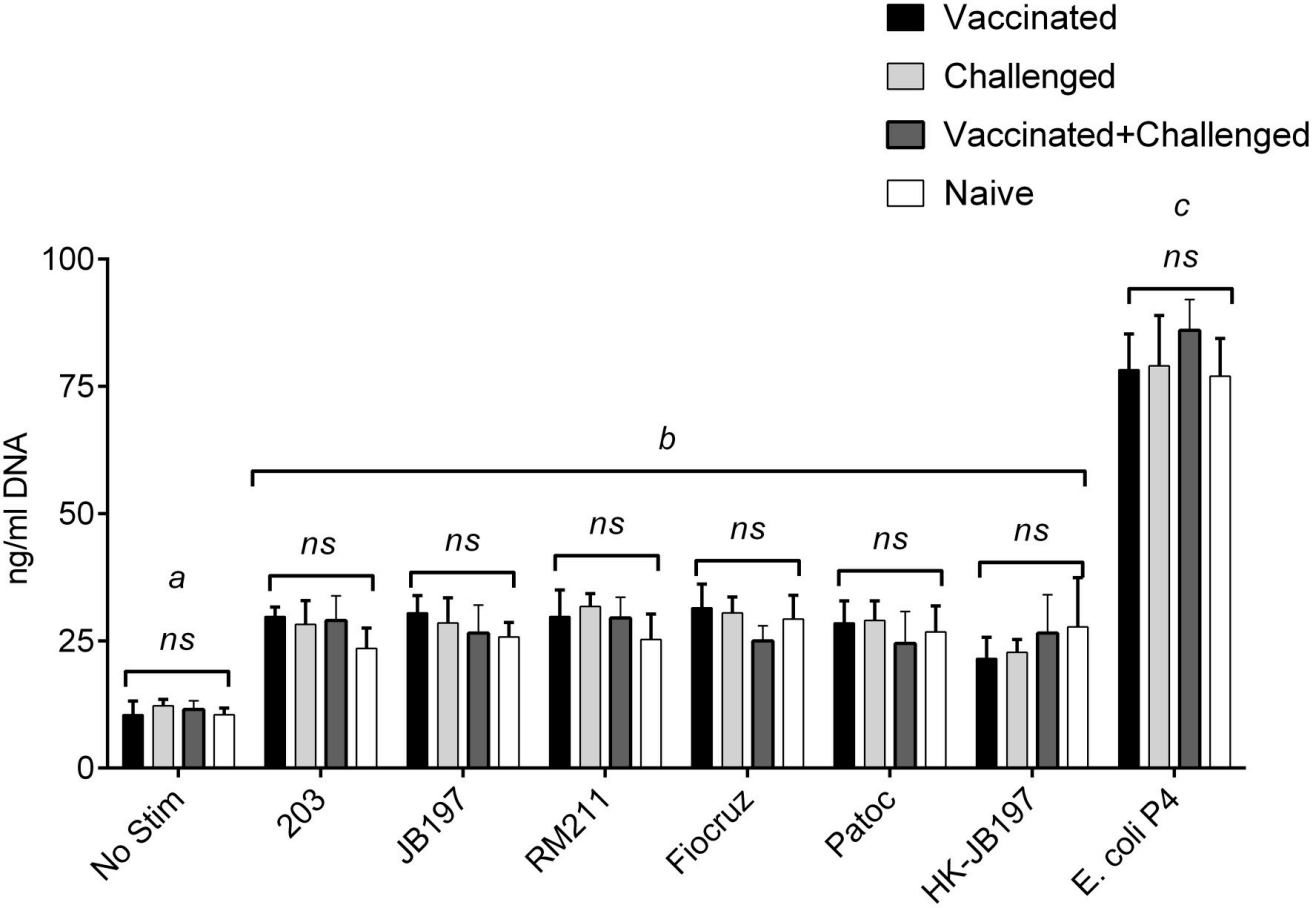
**Quantitation of extracellular DNA as an indication of NET formation by bovine PMNs in the presence of ***Leptospira*** and ***Leptospira*** reactive immune serum**. Following incubation of isolated bovine PMNs in supplemented RPMI 1640 with 20% pooled serum from vaccinated, challenged, vaccinated + challenged, or naïve cattle, with media only (No Stim), *L. borgpetersenii* serovar Hardjo strain 203, *L. borgpetersenii* serovar Hardjo strain JB197, *L. interrogans* serovar Pomona strain RM211, *L. interrogans* serovar Copenhageni strain Fiocruz L1-130, *L. biflexa* strain Patoc, heat-killed *L. borgpetersenii* serovar Hardjo strain JB197, or *E. coli* P4 in 96 well plates, extracellular DNA was released by digestion with Staphylococcal Endonuclease and supernatants assayed using fluormetric DNA quantitation assay (PicoGreen). Results depicted are mean and standard deviation (error bars) of cells from four individual animals assayed in two independent experiments. Leptospira strains (treatments) were compared across serum types (naïve, vaccinated, etc.) were compared using one-way ANOVA and effect of added immune serum was not significant (*p* < 0.05). Treatments were compared to each other by one-way ANOVA with Tukey's multiple comparisons post-test. Treatments with different letters (*a, b, c*) are statistically different from each other but not from treatments with the same letter (*p* < 0.05).

Serum banked and pooled from a previously published study was used as the source for immune serum (Zuerner et al., 2011). The sera pools used in this assay [naïve, vaccinated, vaccinated, and infected (vaccinated + challenged), or naïve-infected (challenged)] had MAT titers to *L. borgpetersenii* serovar Hardjo strains 203 and JB197 that were negative (< 1:12), 1:200, 1:800 and 1:100 for the four groups respectively (Table S1). All serum pools were MAT negative for other *Leptospira* strains. NETosis assay (measurement of extracellular DNA) was performed with the presence of bovine serum from naïve, vaccinated, vaccinated, and infected (vaccinated + challenged), or naïve-infected (challenged) cattle which contained *Leptospira* specific antibody (Zuerner et al., 2011). Addition of bovine anti-leptospiral serum (naïve, Vaccinated, Vaccinated + Challenged, or Challenged) at 20% of the final cell culture media did not alter the response (NET formation) to any individual *Leptospira* strain (Figure 7). Within a PMN treatment with *Leptospira*, there was no significant difference when different immune serum was added (*p* < 0.05) (Figure 7), nor did the overall results of the assay change from the trend depicted in Figure 2, where all *Leptospira* strains induced similar level of NETosis (22.3 to 29.8 ng/ml DNA in culture supernatants) and were significantly different from *E. coli* P4 (with 77.0–86.1 ng/ml DNA in culture supernatant) and all greater than no stimulant (No Stim 10.5–12.25 ng/ml DNA).

### Effect of immune serum on PMN *leptospira* killing

PMN and *Leptospira* incubation was performed as above with the addition of bovine anti-leptospiral serum, at 20% of media. No effect was observed on *Leptospira* survival (Table S2). Growth was consistently observed out to the –6 dilution, same as for *Leptospira* manipulated and incubated under the same conditions, without the presence of bovine PMNs or immune serum.

## Discussion

Recent reports demonstrated human and mouse neutrophils produced NETs in response to pathogenic *Leptospira*, resulting in killing of the *Leptospira* and reduced tissue burdens (Scharrig et al., 2015). Older studies documented *Leptospira* interactions with human and guinea-pig PMNs (McGrath et al., 1984; Wang et al., 1984a). However, infections in these species are more representative of acute leptospirosis, hallmarked by an overt inflammatory reaction and systemic septicemia-like condition. Cattle are a reservoir host of *Leptospira*, demonstrating chronic infection, with colonization limited to a few tissues (reproductive and urinary tracts) and intermittently shed *Leptospira*, infecting herd-mates, humans and other animals (Gamage et al., 2014; Samir et al., 2015). Therefore, it is important to understand the complex interactions of host/*Leptospira* in both types of infection, the acute and chronic/reservoir disease in the proper host. These studies were attempted to evaluate if and how various strains of *Leptospira* would activate bovine PMNs.

One very visual determination of neutrophil or PMN activation is induction of NETosis. Previously NETosis has been demonstrated with *E. coli* P4 and other bacterial pathogens (Lippolis et al., 2006; Aulik et al., 2010). Incubation of bovine PMNs with *Leptospira* strains did induce some NET activation, and while greater than background, it was much less than that with the *E. coli* P4 pathogen or PMA. In contrast to previous reports using *B. burgdorferi*, confirmation of leptospiral structures within NET formations was not observable (Menten-Dedoyart et al., 2012), most likely due size difference, *Leptospira* being half the length and width of *Borrelia* species. Using an assay to measure DNA extruded into the cell culture supernatant, we observed a roughly two-fold increase over background when bovine PMNs were incubated with leptospiral strains. This increase was diminished with the addition of DNase enzyme, further indicating that NETosis was occurring. Again, in contrast to recent publication using human neutrophils (Scharrig et al., 2015), we did not observe a difference in magnitude of response in regards to virulence; the same response was seen for pathogenic and saprophytic strains, and for heat-inactivated organisms. We have confidence overall in the execution of our assays because in our studies, percentages of *E. coli* P4 or PMA stimulated cells undergoing NETosis were consistent with previous reports (Lippolis et al., 2006; Aulik et al., 2010; Wardini et al., 2010). Previously published studies have demonstrated that NETs contain both nucleic acids and histone proteins (Brinkmann et al., 2004; Aulik et al., 2010; Gunderson and Seifert, 2015). Using histone antibody staining as a secondary indication of NET formation, we again observed an increase in the number of PMNs with NETs as compared to background/no stimulant, but less than *E. coli* P4 or PWM mitogen. Taken together, *Leptospira* did induce NETosis in bovine PMNs, just not as robustly as *E. coli* P4 or PMA mitogen.

Stimulation of pattern recognition receptors (PRRs) along with pro-inflammatory cytokines (IFN-γ, IL-1β, and TNF-α) can trigger signaling cascades leading to iNOS production including NF-kB and MAPK (Fang, 2004). Production of nitric oxide (NO) and superoxide ions represent a mechanism of pathogen destruction in activated neutrophils, and production or expression of iNOS has been associated with many inflammatory associated diseases (Mariano et al., 2012). During the “respiratory burst,” superoxide can react with many potential complexes, including nitric oxide to form the reactive nitrogen species (RNS) peroxynitrite (Amulic et al., 2012). Usually produced more from macrophages than neutrophils, RNSs can control as well as contribute to local tissue and vascular inflammation through signaling and other non-antimicrobial functions (Fang, 2004). In the current study, H_2_O_2_ produced by PMN in response to incubation with *Leptospira* strains was increased over no stimulation, but the overall levels were low (0.4–0.6 μM H_2_O_2_) calling into question the biological significance. In a cell free assay, Murgia et al. observed that pathogenic *L. interrogans* strain Hardjoprajitno showed a 2 Log reduction in the presence of 0.06 mM H_2_O_2_ and complete inhibition at 0.34 mM H_2_O_2_ (Troxell et al., 2014). These levels are at least 100 times greater in H_2_O_2_ concentration than was measured in the present study. In contrast, bovine PMNs produced higher levels of NO_2_ in the presence of *E. coli* P4 and significant amounts in the presence of *Leptospira* strains, again, with no difference to virulence or viability. NO_2_, a product of NO and O${}_{2}^{-}$ can react with pathogen targets and effect innate immune pathogen clearance (Lowenstein and Padalko, 2004).

Neutrophil-derived cytokines can play a role in orienting immunity toward Th-1 type responses [production of IL-12, macrophage inflammatory protein (MIP)-1α] (Scapini et al., 2000). Interleukin-8 is produced in large amounts by neutrophils in response to external stimuli and can play a role in the inflammatory recruitment cascade (Hassfurther et al., 1994; Scapini et al., 2000). Neutrophils can also produce TNF-α which can elicit cytokine responses in an endocrine-like feedback loop for both neutrophil and surrounding cells (Walter and Morck, 2002). Production of MIP– 1α, 1β, and 1γ by neutrophils can be chemotactic and stimulatory for monocytes, macrophages, dendritic cells, NK cells, and Th1 lymphocytes (Scapini et al., 2000). After incubation with bacterial lipopolysaccharide, bovine neutrophils have been known to produce inflammatory cytokines including IL-8, IL-1β, and TNF-α (Worku and Morris, 2009). Hamster kidney following infection with virulent *Leptospira*, or hamster peripheral blood mononuclear cells (PBMCs) cultured with *Leptospira*, upregulate TNF-α, IFN-γ, IP-10, IL-10, and IL12p40 (Vernel-Pauillac and Merien, 2006; Lowanitchapat et al., 2010). Stimulation of human cell line THP-1, PBMCs or whole blood with *Leptospira* resulted in production of TNF-α and IL-6 (Goris et al., 2011). In the present study, we observed increases in IL-1β, IL-8, MIP-1α, and TNF-α cytokine gene expression. While increases in pro-inflammatory cytokine gene expression were observed from PMNs incubated with *Leptospira* these were far less that those observed with *E. coli* P4. Interestingly, cytokine expression was also dependent on live leptospires being present as heat-killed organisms did not induce the same magnitude of effect. While not reaching significance, there was a trend for the saprophyte Patoc to induce less cytokine gene transcript than the pathogenic (Hardjo 203 and JB197, Pomona RM211, and Fiocruz) strains used.

Although our data indicated modest evidence of NET activation, production of RNS, and slight increase in pro-inflammatory cytokine gene expression, we originally hypothesized that bovine PMNs could still influence clearance of *Leptospira* by phagocytosis or some other method of bacterial killing. However, any PMN activation had no effect on *Leptospira* viability. We also did not observe killing of non-pathogenic strain of *Leptospira*, whereas others observed that non-pathogenic leptospires were able to be killed by human PMNs (Wang et al., 1984a,b). Using a most-probable-number procedure for quantitating viable numbers of *Leptospira*, they showed that while the pathogenic *Leptospira* became cell associated, they were not killed in the presence of neutrophils and immune serum (Wang et al., 1984a). More recently, Goris et al. showed that human whole blood was able to kill culture-adapted strains, but not host-adapted strains (Goris et al., 2011). Furthermore, THP-1 cells or PMBCs were not able to kill culture- or host- adapted strains (Goris et al., 2011). These differences in reported killing of *Leptospira* by whole blood or isolated cells may be attributed to differences in methods for *Leptospira* culture/viability or host cell type used in assays. We observed no reduction in *Leptospira* viability, including the non-pathogenic saprophyte strain, by bovine PMNs, as measured by dilution culture a more sensitive means of determining most probable numbers for difficult to culture organisms. Assays with *E. coli* P4 showed reductions in viability consistent with neutrophil killing assays, indicated that PMNs were capable of antimicrobial activities (Ermert et al., 2009). This further illustrates differences between acute and chronic/reservoir hosts in response to infection.

To test if opsonization was needed for *Leptospira*-PMN interaction or leptospiral killing, NET quantitation and leptospiral assays were conducted with the inclusion of immune serum. The serum titer or antibody reactivity was determined by MAT, which by its very description requires surface binding antibody. Inclusion of *Leptospira* specific antibodies did not enhance the results over naïve or non-immune serum nor did it change the results, all *Leptospira* strains resulted in similar levels of NETosis. Inclusion of *Leptospira* specific serum did not alter *Leptospira* viability either. While there is a trend for there to be less growth at the higher (−6 and −7 dilutions) for strain 203 in the presence of immune serum, the results are not consistent or striking enough to be of importance. Due to the free-thawing that the serum underwent, and the heat inactivation, it is unlikely that any active complement remained in the serum sample. Overall, current data indicates that clearance of *Leptospira* and *Leptospira* killing by innate cells is complex and may involve active complement, specific antibody and multiple phagocytic cell types (Wang et al., 1984b; Goris et al., 2011).

Differences between species in innate immunity function have been recently reported, illustrating that caution must be used when making assumptions regarding host-pathogen relationships. The differences in spirochete recognition by TLR receptors between mice and humans, and differences between bovine, mice and human TLR2 receptors at the level of amino acid homology, might explain differences in responses through activation of TLR receptors and subsequent inflammatory cascades. Further studies will be needed to confirm that bovine TLR2 does not recognize *Leptospira* antigens. This emphasizes the importance of studying pathogen-host interactions in native hosts.

Unlike other spirochetes such as *Treponema* or *Borrelia, Leptospira* species possess lipopolysaccharide (LPS) as part of their outer membrane (Nahori et al., 2005). Typically LPS is recognized by the pattern recognition receptor (PRR) toll-like receptor TLR4, leading to pro-inflammatory cytokine, and chemokine responses (Nahori et al., 2005). Recently it has been shown that *Leptospira* LPS is recognized by porcine, mouse and human TLR2, and neutrophils from these species, express surface TLR2 (Nahori et al., 2005; Thomas and Schroder, 2013; Guo et al., 2015). While bovine PMNs do express TLR2 and TLR4 (Swain et al., 2014), there are species differences in TLR structure that may impact functionality with certain bacterial moieties. Bovine TLR2 shares 77 and 65% homology at the amino acid level with human and murine TLR2, and 72 and 65% homology for TLR4 (Werling and Jungi, 2003). Ligation of bacteria to PRRs may promote neutrophil survival (increase in lifespan), ROS production (increase in myeloperoxidase activity), NET formation, and cytokine production, all of which may be detrimental to the bacterial pathogen (Yipp et al., 2012; Thomas and Schroder, 2013). In contrast to humans, the ability of mice to recognize *Leptospira* LPS and Lipid A through TLR4 and MyD88 independent mechanisms, may explain some of the observed differences between humans and mice regarding susceptibility to infection (Fraga et al., 2011).

## Conclusion

Our data demonstrates *Leptospira* strains activate circulating PMNs of cattle greater than unstimulated cells, but not to the same levels of activation seen with another pathogen, a mastitis inducing strain of *E. coli*. Furthermore, this modest activation had no impact on the viability of the leptospires. Important to note, whether it was PMN activation or *Leptospira* survival, results were the same regardless of leptospiral strain used: cattle-host adapted, acute pathogenic or saprophytic. We speculate that nuances in bovine pattern recognition receptors, specifically TLR2, may explain our observation in cattle as compared to reports of activation of neutrophils by *Leptospira* in other species (human and mouse). This corresponds to phenotypic observations of wild rodents being chronic reservoirs of *Leptospira* carriage while humans are susceptible to acute but rarely chronic forms of the disease (Ganoza et al., 2010; De Silva et al., 2014; Loffler et al., 2014; Matsui et al., 2016). In comparison, cattle are susceptible to chronic infection with *Leptospira* and this correlates with the clinical presentation of a stealth disease residing in kidney and reproductive tissues. Furthermore, the results from this study, using bovine PMNs, contrast with those reported elsewhere using neutrophils or PMNs from humans and other acute hosts. These results show that differences in disease state (chronic or acute) may be host associated as well as *Leptospira* strain or specific. Continued study in the reservoir host is needed to fully understand the host-pathogen relationship.

## Author contributions

DA and JW conceptualized original experiments. JW came up with overall experimental design. JW, AF, RH performed the experiments and were responsible for data analysis. JW, AF, RH, SO and DA all contributed to critical evaluation of the data, manuscript preparation and final editing of the manuscript.

## Funding

This work was performed by USDA employees in the fulfillment of their regularly assigned duties.

### Conflict of interest statement

The authors declare that the research was conducted in the absence of any commercial or financial relationships that could be construed as a potential conflict of interest.
