# Supplementary material for: Interaction of Bovine Peripheral Blood Polymorphonuclear Cells and Leptospira Species; Innate Responses in the Natural Bovine Reservoir Host
Source: Front Microbiol. 2016 Jul 19;7:1110. doi: 10.3389/fmicb.2016.01110 (PMC4949235; doi:10.3389/fmicb.2016.01110)
Supplement: Supplementary file 1 [file Table1.docx]

| Table S1: Microscopic Agglutination Titers (MAT) of the four serum pools against *Leptospira* strains used in this study. | | | | | |
| --- | --- | --- | --- | --- | --- |
| Pooled Sera | 203 | JB197 | RM211 | Fiocruz | Patoc |
| Naive | *Neg^*^* | *Neg* | *Neg* | *Neg* | *Neg* |
| Vaccinated | 1:200 | 1:200 | *Neg* | *Neg* | *Neg* |
| Challenged | 1:800 | 1:800 | *Neg* | *Neg* | *Neg* |
| Vaccinated + Challenged | 1:100 | 1:100 | *Neg* | *Neg* | *Neg* |
| *^*^Neg: negative*, titer <1:12. | | | | | |
