# Supplementary material for: Interaction of Bovine Peripheral Blood Polymorphonuclear Cells and Leptospira Species; Innate Responses in the Natural Bovine Reservoir Host
Source: Front Microbiol. 2016 Jul 19;7:1110. doi: 10.3389/fmicb.2016.01110 (PMC4949235; doi:10.3389/fmicb.2016.01110)
Supplement: Supplementary file 2 [file Table2.docx]

| Table S2: *Leptospira* killing by bovine PMNs with incubation of immune serum. | | | | | | | | |
| --- | --- | --- | --- | --- | --- | --- | --- | --- |
| Serum | Strain | Culture Result^1^ | | | | | | |
|  |  | -1^2^ | -2 | -3 | -4 | -5 | -6 | -7 |
| Naïve | JB197 | 4/4 | 4/4 | 4/4 | 4/4 | 3/4 | 2/4 | 2/4 |
| Naïve | 203 | 4/4 | 4/4 | 4/4 | 4/4 | 4/4 | 2/4 | 2/4 |
| Naïve | RM211 | 4/4 | 4/4 | 4/4 | 4/4 | 4/4 | 3/4 | 2/4 |
| Naïve | Fiocruz | 4/4 | 4/4 | 4/4 | 4/4 | 4/4 | 3/4 | 2/4 |
| Naïve | Patoc | 4/4 | 4/4 | 4/4 | 4/4 | 3/4 | 3/4 | 2/4 |
| Vaccinated | JB197 | 4/4 | 4/4 | 4/4 | 4/4 | 3/4 | 3/4 | 2/4 |
| Vaccinated | 203 | 4/4 | 4/4 | 4/4 | 4/4 | 3/4 | 2/4 | 2/4 |
| Vaccinated | RM211 | 4/4 | 4/4 | 4/4 | 4/4 | 4/4 | 4/4 | 4/4 |
| Vaccinated | Fiocruz | 4/4 | 4/4 | 4/4 | 4/4 | 4/4 | 4/4 | 3/4 |
| Vaccinated | Patoc | 4/4 | 4/4 | 4/4 | 4/4 | 3/4 | 2/4 | 1/4 |
| Challenged | JB197 | 4/4 | 4/4 | 4/4 | 4/4 | 4/4 | 2/4 | 2/4 |
| Challenged | 203 | 4/4 | 4/4 | 4/4 | 3/4 | 2/4 | 2/4 | 1/4 |
| Challenged | RM211 | 4/4 | 4/4 | 4/4 | 4/4 | 4/4 | 3/4 | 3/4 |
| Challenged | Fiocruz | 4/4 | 4/4 | 4/4 | 4/4 | 4/4 | 4/4 | 2/4 |
| Challenged | Patoc | 4/4 | 4/4 | 4/4 | 4/4 | 3/4 | 3/4 | 2/4 |
| Vaccinated + Challenged | JB197 | 4/4 | 4/4 | 4/4 | 4/4 | 3/4 | 2/4 | 1/4 |
| Vaccinated + Challenged | 203 | 4/4 | 4/4 | 4/4 | 4/4 | 2/4 | 2/4 | 2/4 |
| Vaccinated + Challenged | RM211 | 4/4 | 4/4 | 4/4 | 4/4 | 4/4 | 3/4 | 3/4 |
| Vaccinated + Challenged | Fiocruz | 4/4 | 4/4 | 4/4 | 4/4 | 4/4 | 3/4 | 3/4 |
| Vaccinated + Challenged | Patoc | 4/4 | 4/4 | 4/4 | 4/4 | 2/4 | 2/4 | 1/4 |
| No Serum or Neutrophils | JB197 | +^3^ | + | + | + | + | + | - |
| No Serum or Neutrophils | 203 | + | + | + | + | + | + | + |
| No Serum or Neutrophils | RM211 | + | + | + | + | + | + | - |
| No Serum or Neutrophils | Fiocruz | + | + | + | + | + | + | - |
| No Serum or Neutrophils | Patoc | + | + | + | + | + | + | - |
| ^1^number of tubes with growth in dilution series/total number of replicate tubes (representing cells from 4 individual cows). Shaded cells highlight those experimental parameters (immune serum and strain) that resulted in 50% reduction in number of tubes with leptospiral growth.  ^2^tube dilution in series.  ^3^Only 1 replicate/tube per experiment, manipulation and incubation of *Leptospira* in absence of PMNs for measurement of viability during experimental procedure. Data presented is representative of duplicate experiments. | | | | | | | | |
